# 2D high resolution vs. 3D whole heart myocardial perfusion cardiovascular magnetic resonance

**DOI:** 10.1093/ehjci/jeab103

**Published:** 2021-06-28

**Authors:** Muhummad Sohaib Nazir, Joy Shome, Adriana D M Villa, Matthew Ryan, Ziyan Kassam, Reza Razavi, Sebastian Kozerke, Tevfik F Ismail, Divaka Perera, Amedeo Chiribiri, Sven Plein

**Affiliations:** 1 School of Biomedical Engineering and Imaging Sciences, King’s College London, St Thomas’ Hospital, 4th Floor Lambeth Wing, St Thomas’ Hospital, Westminster Bridge Road, London SW1 7EH, UK; 2 British Heart Foundation Centre of Excellence and National Institute for Health Research Biomedical Research Centre at the School of Cardiovascular Medicine and Sciences, Kings College London, London, UK; 3 Institute for Biomedical Engineering, University and ETH Zurich, Zurich, Switzerland; 4 Department of Biomedical Imaging Science, Leeds Institute of Cardiovascular and Metabolic Medicine, University of Leeds, Leeds Teaching Hospitals NHS Trust, Leeds, UK

**Keywords:** myocardial perfusion, myocardial ischaemic burden, coronary artery disease

## Abstract

**Aims:**

Developments in myocardial perfusion cardiovascular magnetic resonance (CMR) allow improvements in spatial resolution and/or myocardial coverage. Whole heart coverage may provide the most accurate assessment of myocardial ischaemic burden, while high spatial resolution is expected to improve detection of subendocardial ischaemia. The objective of this study was to compare myocardial ischaemic burden as depicted by 2D high resolution and 3D whole heart stress myocardial perfusion in patients with coronary artery disease.

**Methods and results:**

Thirty-eight patients [age 61 ± 8 (21% female)] underwent 2D high resolution (spatial resolution 1.2 mm^2^) and 3D whole heart (in-plane spatial resolution 2.3 mm^2^) stress CMR at 3-T in randomized order. Myocardial ischaemic burden (%) was visually quantified as perfusion defect at peak stress perfusion subtracted from subendocardial myocardial scar and expressed as a percentage of the myocardium. Median myocardial ischaemic burden was significantly higher with 2D high resolution compared with 3D whole heart [16.1 (2.0–30.6) vs. 13.4 (5.2–23.2), *P* = 0.004]. There was excellent agreement between myocardial ischaemic burden (intraclass correlation coefficient 0.81; *P* < 0.0001), with mean ratio difference between 2D high resolution vs. 3D whole heart 1.28 ± 0.67 (95% limits of agreement −0.03 to 2.59). When using a 10% threshold for a dichotomous result for presence or absence of significant ischaemia, there was moderate agreement between the methods (κ = 0.58, *P* < 0.0001).

**Conclusion:**

2D high resolution and 3D whole heart myocardial perfusion stress CMR are comparable for detection of ischaemia. 2D high resolution gives higher values for myocardial ischaemic burden compared with 3D whole heart, suggesting that 2D high resolution is more sensitive for detection of ischaemia.

## Introduction

Dynamic contrast-enhanced stress cardiovascular magnetic resonance (CMR) myocardial perfusion imaging has a high diagnostic accuracy for the detection of significant coronary artery disease (CAD)^1^ and is recommended in European Society of Cardiology (ESC) guidelines for patients with chronic coronary syndromes.^2^ Several methods have been proposed for stress perfusion CMR, with a range of pulse sequences, spatial and temporal resolutions, cardiac coverage, and contrast regimes. In clinical practice, stress myocardial perfusion CMR is typically acquired in three 2D slices covering the 16 myocardial segments, but does not achieve full myocardial coverage. Nascent 3D techniques can achieve whole heart coverage, which may allow more accurate estimation of myocardial ischaemic burden. Whole heart CMR has shown to have a good agreement with single-photon emission computed tomography (SPECT) for assessment of ischaemic burden.^3^ In contrast, high spatial resolution perfusion CMR may be more sensitive for detection of subendocardial ischaemia.^4^

In a previous pilot study, 2D and 3D stress myocardial perfusion CMR were compared in patients with suspected CAD and demonstrated no overall significant bias although there was a large variation between measurements.^5^ In addition, most patients had low ischaemic burden (typically <10–15%) and the two stress perfusions were performed on different days which may have introduced physiological variation. In this study, we sought to undertake 2D and 3D stress perfusions in the same scan on the same day in a larger cohort of patients and in a broader range of ischaemic burden including patients with multivessel CAD. We aimed to assess the presence of ischaemia and the magnitude of difference in myocardial ischaemic burden of 2D high resolution stress vs. 3D whole heart myocardial perfusion CMR in patients with CAD.

## Methods

### Study population

Fifty-four patients with known or suspected CAD listed for an invasive coronary angiography (ICA) as part of routine clinical care were recruited. Patients were recruited either before or after ICA. For the latter, patients were only included if no revascularization was undertaken. Exclusion criteria were contraindication to MRI, previous coronary artery bypass graft (CABG) surgery, recent myocardial infarction (<6 months), unstable angina, or contraindication to adenosine or gadolinium contrast. Patients were advised to refrain from caffeine for 24 h prior to CMR. The study was approved by the National Research Ethics Service (15/NW/0778) with written informed consent obtained from all patients.

### CMR protocol

CMR scans were performed on a 3-T MRI scanner (Achieva TX, Philips Healthcare, Best, The Netherlands) with dual-source parallel radiofrequency transmission technology and a 32-channel cardiac coil. Cine imaging for biventricular volumes and function was acquired with a balanced steady-state free precession (SSFP) cine sequence as recommended in guidelines.^6^ Patients underwent two contrast-enhanced stress perfusion imaging in the same scan; 2D high resolution imaging with three slices in the short-axis orientation; and 3D whole heart imaging with contiguous slices. The two stress perfusions were separated by a minimum of 15 min to allow for contrast washout from the first contrast injection and the order of stress perfusion imaging was randomized.

Stress perfusion images were acquired following administration of intravenous adenosine at 140 µg/kg/min for a minimum of 3 min. An adequate stress response was determined by a decrease of 10 mmHg systolic blood pressure and/or a 10% increase in heart rate and typical symptoms and in cases in which there was a lack of response, the dose of adenosine was increased to 175 and 210 µg/kg/min.^7^ A single intravenous bolus of 0.075 mmol/kg body weight of gadobutrol (Gadovist, Bayer, Germany) was injected at 4 mL/s followed by 25 mL normal saline flush by a power injector (Spectris Solaris^®^ EP, MEDRAD, Inc., USA). For both stress perfusions, patients were asked to breath hold at point of contrast arrival to the myocardium.

For 3D whole heart myocardial perfusion imaging, the 3D volume was planned from the systolic frame of the four- and two-chamber cine with exclusion of the left ventricular outflow tract in the three-chamber cine. A 3D spoiled turbo gradient echo sequence was utilized, with typical parameters: TR/TE/flip angle 1.86 ms/0.7 ms/11°; saturation prepulse delay 150 ms; linear k-space encoding; 70% partial Fourier acquisition in two dimensions; typical field of view 350 mm × 350 mm^2^; 10-fold *k-t* acceleration and 11 training profiles leading to a net acceleration of 7.0; typical acquisition window 192–230 ms, *k-t* PCA reconstruction; reconstructed to 12 contiguous slices with voxel size 2.3 mm × 2.3 mm × 5 mm^3^.

For 2D high resolution imaging, three slices were planned from the base, mid, and apex using the ‘3 of 5’ approach, planned in systole. A turbo gradient echo sequence was utilized with typical parameters: TR/TE/flip angle 2.6 ms/0.9 ms/20°; saturation prepulse delay 120 ms; linear *k-*space encoding; no partial Fourier acquisition; typical field of view 340 mm × 340 mm^2^; 5-fold *k-t* acceleration with 11 interleaved training profiles; typical acquisition window 100–134 ms, *k-t* PCA reconstruction; reconstructed to 3 non-contiguous slices with voxel size 1.2 mm × 1.2 mm × 8 mm^3^.

Late gadolinium-enhanced (LGE) imaging was performed 10 min following top up to 0.2 mmol/kg of gadolinium contrast from the second contrast injection. A T1-weighted segmented inversion recovery gradient echo was used (typical sequence parameters: TR/TE/flip angle 3.9 ms/2.0 ms/25°; pixel size 1.35 mm × 1.35 mm^2^). LGE images were acquired in standard long-axis planes and also the entire short-axis views with 8-mm slice thickness and no slice gap.

### Image analysis

Image reconstruction of the 3D perfusion sequences was performed using ReconFrame (Gyrotools LLC, Zurich, Switzerland). 3D whole heart and 2D high resolution myocardial perfusion images were anonymized and randomly presented to two experienced CMR readers with 7 and 6 years’ experience in CMR, respectively. Image quality was graded on a four-point scale (0 = poor image quality and non-diagnostic, 1 = major artefact present but not limiting diagnosis, 2 = minor artefact present but not limiting diagnosis, and 3 = excellent).

The presence and location of ischaemia were recorded using a 16-segment model. Myocardial ischaemia was visually quantified on DICOM images on a workstation using CVI-42 software version 5.12.2 (Circle CVI, Calgary, Canada). Perfusion defects were determined visually based on the presence of a subendocardial or transmural defect, which persisted for more than five dynamic phases, in areas that corresponded to a coronary artery territory, and exhibited delayed contrast enhancement compared to other myocardial segments in the same slice. The perfusion defect was contoured at peak stress perfusion and expressed as a percentage of the total myocardial volume in the perfusion images to derive the ‘total perfusion defect’. LGE was considered to be present if seen in long-axis and short-axis views. In order to quantify the amount of LGE, the endocardial and epicardial borders were outlined and then manual planimetry of areas of high signal intensity on a background of nulled myocardium was performed in each slice. Following summation of LGE areas, these were then multiplied by the slice thickness. This manual planimetry approach was employed, as there is no agreed consensus on method of LGE quantification in recent international guidelines.^8^ The entire process was then repeated in 10 random cases, 2 months later to determine intraobserver reproducibility.

### Coronary angiography

Patients underwent ICA at Guy’s and St Thomas’ Hospital, London. Quantitative coronary angiography (QCA) was performed retrospectively on X-ray coronary angiography images offline (Medcon Ltd., Tel Aviv, Israel) by an experienced observer with more than 5 years’ experience in coronary angiography. Significant CAD was defined as vessel with FFR value of ≤0.80, and in those vessels in which FFR was not undertaken was defined by a coronary luminal stenosis >70% on QCA. The invasive measurement of ischaemic burden, the British Cardiovascular Intervention Society (BCIS) Duke’s Jeopardy score, was calculated as previously described.^9^

### Statistical analyses

Statistical analyses were performed using SPSS Statistics 24 (IBM, Armonk, NY, USA). Normality was assessed using Shapiro–Wilk tests. Results are expressed as mean ± standard deviation unless otherwise specified. Student *t*-tests were used to compare means for parametric data, and Wilcoxon signed-rank test to compare image quality. Agreement of quantitative ischaemic burden and perfusion defect were analysed using intraclass correlation coefficients (ICCs) and compared using Wilcoxon signed-rank test and Bland and Altman analysis. For Bland and Altman analysis, in situations in which there was a non-random variation in differences (e.g. an increase in differences of measurements with increasing mean), a ratio transformation of values was performed.^10^ Presence of significant ischaemia was compared using Cohen’s Kappa coefficient. Interobserver and intraobserver agreements were assessed with Cohen’s Kappa coefficient for categorical data and coefficients of variation (CoV) and ICC for continuous data. Two-tailed values of *P* < 0.05 were considered statistically significant.

## Results

### Baseline characteristics

**Figure 1:**
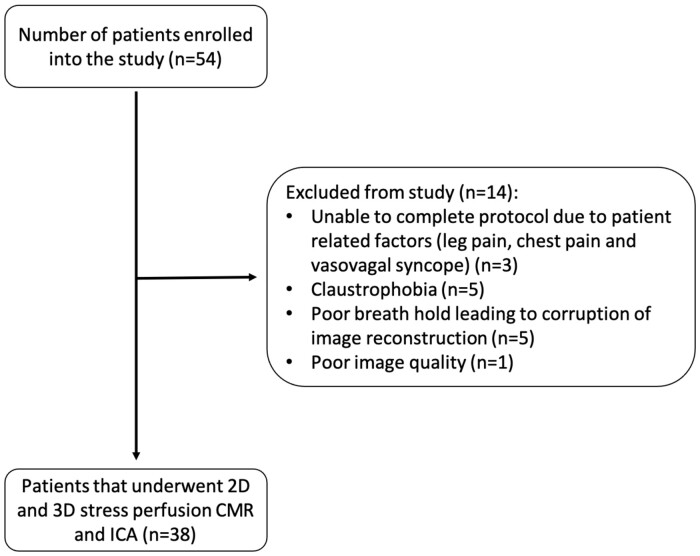
A **f**lowchart which demonstrates patient recruitment and exclusion from the study.

Fifty-four eligible patients were considered for enrolment and 38 patients were recruited after exclusions (*Figure 1*). All patients underwent ICA with a median of 9 days (interquartile range 3–16) of the CMR scan. Patient demographics are summarized in *Table 1* and the distribution of CAD is provided in *Table 2*.

**Table 1:** Baseline demographics and patient characteristics (*n* = 38)

Demographics and risk factors, *n* (%)	
Age (years)	61 ± 8
Gender	8 female (21%)
BMI (kg/m^2^)	28 ± 4
Hypertension	24 (63%)
Hyperlipidaemia	24 (63%)
Smoking history	21 (55%)
Diabetes mellitus	5 (13%)
Family history IHD	16 (42%)
Previous myocardial infarction	5 (13%)
Previous PCI	3 (8%)

MI, myocardial infarction; PCI, percutaneous coronary intervention; IHD, Ischaemic Heart Disease.

**Table 2:** Distribution of coronary artery disease

No significant coronary artery disease	10 (26%)		
Single vessel	14 (37%)		
Two vessels	12 (32%)		
Three vessels	2 (5%)		
		Stenosis	FFR
Left anterior descending artery	21 (55%)	70.8 ± 23.2	0.80 ± 0.06
Left circumflex artery	10 (26%)	70.9 ± 21.2	0.90 ± 0.09
Right coronary artery	13 (34%)	71.0 ± 24.1	0.88 ± 0.09

Significant vessel disease was defined by quantitative coronary angiographic >70% stenosis, or by fractional flow reserve of ≤0.80 for intermediate lesions. Quantitative coronary angiography. Data are presented as *n* (%) or mean ± standard deviation.

FFR, fractional flow reserve; MI, myocardial infarction; PCI, percutaneous coronary intervention.

### Image quality

Data from 6 of the initial 54 patients were not included in the study due to non-diagnostic image quality. Five of these related to 3D datasets that were degraded by motion that corrupted the reconstruction process. In the 38 diagnostic datasets, there was a trend for superior image quality with 2D high resolution median score (interquartile range) 3 (2–3) compared to 3D whole heart [2 (2–3)], although this was not statistically significant (*P* = 0.09).

### Haemodynamic data

For 2D high resolution stress, heart rate increased from 63 ± 10 to 90 ± 16 bpm (*P* < 0.0001) and rate pressure product (RPP) increased from 8366 ± 1897 to 11695 ± 2644 mmHg min^−1^ (*P* < 0.0001) for rest and stress, respectively. For 3D whole heart stress, heart rate increased from 63 ± 10 to 91 ± 17 bpm (*P* < 0.0001) and RPP increased from 8463 ± 1608 to 11551 ± 2563 mmHg min^−1^ (*P* < 0.0001). There was no significant difference between rest heart rate, stress heart rate, blood pressure, or RPP between the two stress perfusions.

### Ischaemic burden

Ischaemia (defined by a perfusion defect in the absence of corresponding LGE or artefact) was present in 29/38 (76%) of patients with 3D whole heart and in 27/38 (71%) with 2D high resolution perfusion imaging. There was excellent agreement between 3D whole heart and 2D high resolution myocardial ischaemic burden with ICC 0.81 (*P* < 0.0001). The median myocardial ischaemic burden (%) was significantly higher with 2D high resolution compared to 3D whole heart [16.1% (2.0–30.6) vs. 13.4% (5.2–23.2), *P* = 0.004]. The mean ratio difference between 2D high resolution vs. 3D whole heart was 1.28 ± 0.67 (95% limits of agreement −0.03 to 2.59).

On subgroup analysis, the mean ratio of difference of myocardial ischaemic burden between 2D high resolution vs. 3D whole heart for patients with no significant CAD was 1.29 ± 1.31 (95% limits of agreement −1.26 to 3.86), single-vessel disease 1.20 ± 0.31 (95% limits of agreement 0.60–1.81), two-vessel disease 1.38 ± 0.54 (95% limits of agreement 0.33–2.42), and three-vessel disease 1.74 ± 0.71 (95% limits of agreement 0.36–3.13).

**Figure 2:**
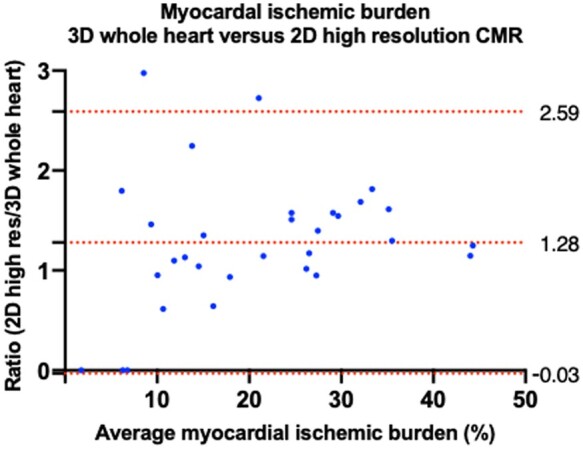
Bland–Altman plot of myocardial ischaemic burden measured with 3D whole heart and 2D high resolution perfusion imaging. Dashed lines from top to bottom indicate the upper 95% limits of agreement, mean bias, and lower 95% limits of agreement.

As shown in the Bland–Altman plot in *Figure 2*, there was a trend for higher myocardial ischaemic burden with 2D high resolution with greater ischaemic burden, particularly over 20%. The mean ratio of difference of myocardial ischaemic burden between 2D high resolution vs. 3D whole heart for patients was greater with patients with a mean ischaemic burden of >20% compared with <20% [1.50 ± 0.46 (95% limits of agreement 0.61–2.39) vs. 1.12 ± 0.76 (95% limits of agreement −0.37 to 2.61)]. When using a 10% threshold for a dichotomous result for presence or absence of significant ischaemia, there was moderate agreement between the two methods (*κ* = 0.58, *P* < 0.0001). There was an excellent intraobserver reproducibility for myocardial ischaemic burden with ICC 0.87 and CoV 4.5%.

### Total perfusion defect

**Figure 3:**
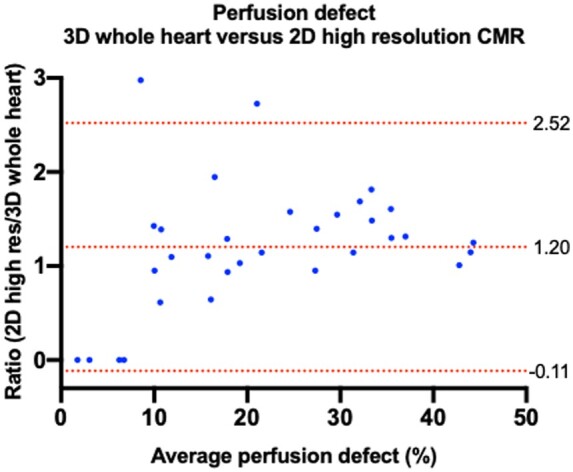
Bland–Altman plot of perfusion defect measured with 3D whole heart and 2D high resolution perfusion imaging. Dashed lines from top to bottom indicate the upper 95% limits of agreement (2.52), mean bias (1.20), and lower 95% limits of agreement (−0.12).

There was an excellent agreement between 3D whole heart and 2D high resolution myocardial ischaemic burden ICC = 0.95 (*P* < 0.0001). The median myocardial perfusion defect (%) was significantly higher with 2D high resolution vs. 3D whole heart [18.4 (2.0-35.4) vs. 15.3 (8.4-23.9), *P* = 0.007]. The mean ratio difference between 2D high resolution vs. 3D whole heart was 1.20 ± 0.67 (95% limits of agreement −0.11 to 2.52). As with the findings for the myocardial ischaemic burden, and shown in *Figure 3*, there was a trend for a higher perfusion defect with 2D high resolution imaging with greater mean ischaemic burden, particularly over 20%.

When using a 10% threshold for a dichotomous result for the presence or absence of significant amount of ischaemia, there was moderate agreement between the two methods (*κ* = 0.56, *P* < 0.0001).

### Comparison with invasive Duke’s Jeopardy Score

Compared with BCIS Duke’s jeopardy score derived from ICA for assessment of myocardial ischaemic burden, there was moderate correlation with 3D ischaemic burden (*r*_s_ = 0.53), 3D perfusion defect (*r*_s_ = 0.59), 2D ischaemic burden (*r*_s_ = 0.55), and 2D perfusion defect (*r*_s_ = 0.58).

## Discussion

In this first head to head study under near identical haemodynamic conditions, we identified that 2D high resolution estimates a higher myocardial ischaemic burden compared to 3D whole heart myocardial perfusion stress CMR, suggesting that 2D high resolution is more sensitive for the detection of ischaemia. This finding was more apparent with increasing ischaemic burden, particularly over 20%. Furthermore, there was only a moderate agreement between the two methods when using a 10% dichotomous threshold for ischaemia.

### Myocardial ischaemic burden

The assessment of ischaemic burden has been of much interest following a large observational study which has suggested a 10% cut-off to guide revascularization or medical therapy in CAD patients.^11^ In the substudy of the COURAGE trial, which used whole heart SPECT imaging, patients who had a reduction in serial ischaemia, had a lower risk of death or myocardial infarction.^12^ The 10% cut-off is commonly extrapolated to other non-invasive imaging tests, such as CMR, which have different imaging properties (such as contrast agent, acquisition method, temporal, and spatial resolution). Importantly, only three slices of the heart are acquired with standard stress myocardial perfusion CMR, which may underestimate myocardial ischaemic burden.^3^ Conversely, 2D high resolution may detect a higher amount of ischaemia, particularly in the subendocardium.^4^ Furthermore, there has been much debate on the value of non-invasive methods to detect ischaemia.^13^

Previous studies have compared myocardial ischaemic burden between different imaging modalities (*Table 3*). In one study (*n* = 45) that compared 3D whole heart myocardial perfusion CMR to SPECT, there was a small bias of 0.62% although wide limits of agreement.^3^ In the same study, a simulated 2D acquisition of the 3D images showed lower ischaemic burden compared to 3D whole heart (5.7% vs. 6.8%). The authors speculated that the lower ischaemic burden with 2D was a consequence of fewer slices that had detected ischaemia leading to an underestimation. However, as acknowledged by the authors, there were limitations in this approach since the extrapolated 2D slices were all acquired during systole, unlike standard 2D perfusion imaging, where slices are acquired at different points of the cardiac cycle. Given the wide limits of agreement, it was not conclusive as to whether ischaemic burden could be used interchangeably.

**Table 3:** Previous comparative studies of myocardial ischaemic burden

Study	Imaging technique	*n*	Comparison of ischaemic burden (inplane spatial resolution)		Difference	Correlation	Bias (95% limits of agreement)
Jogiya *et al*.^3^	3-T CMR and SPECT	38	3D whole heart CMR (2.3 mm^2^)	SPECT	Mean ischaemic burden 6.8% (CMR) vs. 7.5% (SPECT)	*r*_s_ = 0.70; *P* = 0.82	–0.62% (95% LOA –14.3 to 13.1%)
Motwani *et al*.^4^	1.5-T CMR	35	2D High resolution (1.6 mm^2^)	Standard resolution (2.5 mm^2^)	20.1 + 7.7 vs. 11.9 + 9.4, *P* < 0.0001		
	CMR only		3D whole heart CMR	2D (3 slices simulated from 3D acquisition)	Mean ischaemic burden 6.8% (3D CMR) vs. 5.7% (simulated 2D)	*r*_s_ = 0.97	
McDiamard *et al*.^5^	3-T CMR	27	3D whole heart (2.3 mm^2^)	2D high resolution (1.6 mm^2^)	4.3 + 5.2 (2D high resolution) vs. 4.1 + 4.9% (3D whole heart), *P* = 0.81	*r* = 0.72; *P* < 0.001	−0.17% (95% LOA −7.5 to 7.2%)
Sharif *et al*.^14^	SPECT total perfusion defect	651	Simulated 3 Slice SPECT	Whole heart SPECT		*r* ^2^ = 0.93, *P* < 0.001	−1.19% (95% LOA −6.65% to 4.27%)

CMR, cardiovascular magnetic resonance; LOA, limits of agreement; SPECT, single-photon emission computed tomography.

In another study (*n* = 35), there was a greater extent of ischaemia detected using high resolution compared to standard resolution 2D myocardial perfusion in patients with angiographically defined three-vessel disease.^4^ This is most likely explained from the better identification of subendocardial perfusion defects with higher spatial resolution. We also obtained similar conclusions in this study, although we compared to a 3D whole heart technique, which has comparatively lower spatial resolution.

Undertaking two stress perfusions is challenging and has logistical challenges and therefore has limited most of the previous study sample sizes. In a retrospective simulation study (*n* = 651), total perfusion defect of whole heart was compared to three slices simulated from SPECT data.^14^ The authors found that three slice imaging underestimated ischaemic burden by a small bias of −1.19% with 95% limits of agreement −6.7% to 4.3%.^14^ These are not unexpected findings as imaging a reduced area of the myocardium will undoubtedly underreport areas of ischaemia. However, the findings are not completely interchangeable to our current study, since SPECT has a lower inplane spatial resolution compared to both 3D and 2D CMR techniques employed in this study, and this study used the same resolution for whole heart SPECT and three slice SPECT data.

In a previous study (*n* = 27) similar to the current one, no significant difference in ischaemic burden was observed between 2D high resolution and 3D whole heart CMR, with a small overall bias −0.17% and also with wide 95% limits of agreement of −7.5 to 7.2%.^5^ Interestingly, when using a 10% threshold for ischaemia to categorize significant myocardial ischaemic burden, there was only fair agreement (κ = 0.29). In contrast to this study, the two perfusion scans were performed with an interval of 17 ± 38 days. In addition, the range of ischaemic burden was typically <15%. Our study adds to the literature as we investigated a larger cohort of patients with a wider range of ischaemic burden and found that 2D high resolution does not underestimate ischaemic burden, and there is an approximate 28% greater myocardial ischaemic burden compared to 3D whole heart.

**Figure 4:**
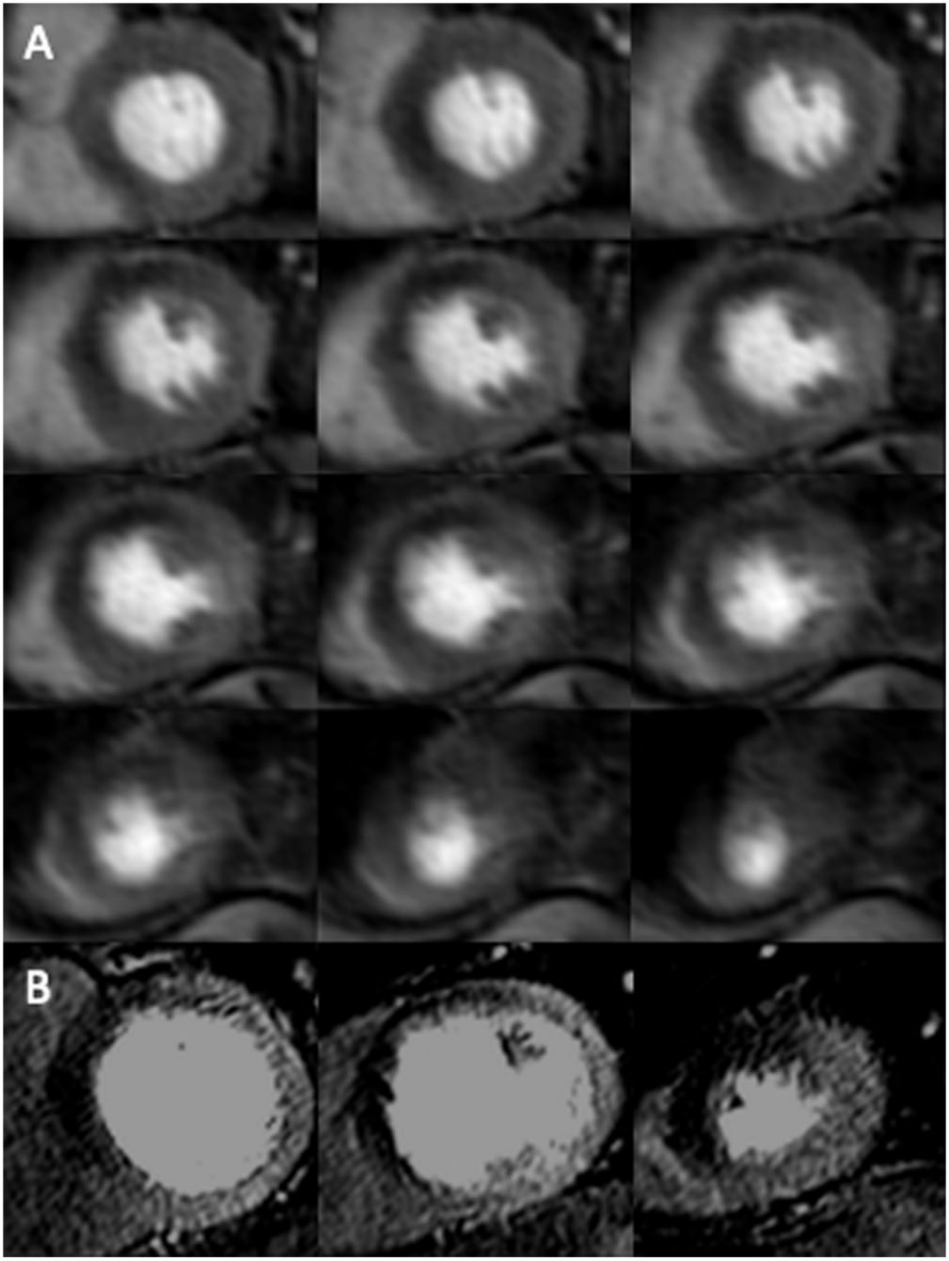
Images at peak myocardial enhancement during adenosine stress perfusion for 3D whole heart (*A*: top to bottom and left to right, from base to apex) and 2D high resolution (*B*: left to right from base to apex). Myocardial ischaemic burden was measured as 23.9% (3D whole heart) vs. 40.3% (2D high resolution). There was no late gadolinium enhancement. Invasive coronary angiography demonstrated an occluded proximal LAD and significant LCx disease.

**Figure 5:**
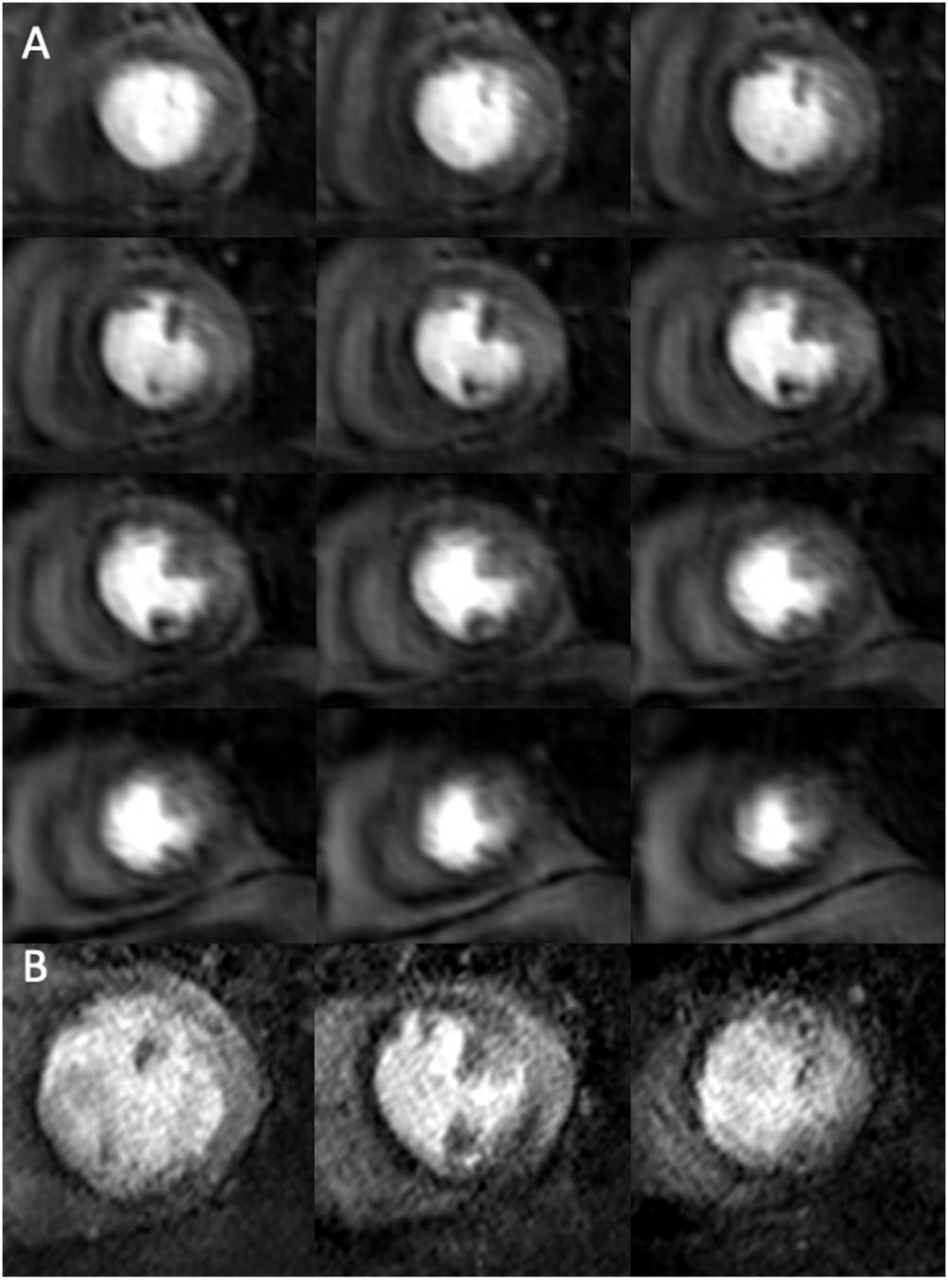
Images at peak myocardial enhancement during adenosine stress perfusion for 3D whole heart (*A*: top to bottom and left to right, from base to apex) and 2D high resolution (*B*: left to right from base to apex). Myocardial ischaemic burden was 39.4% (3D whole heart) vs. 49.2% (2D high resolution). There was no late gadolinium enhancement. Invasive coronary angiography demonstrated significant LAD and LCx disease.

**Figure 6:**
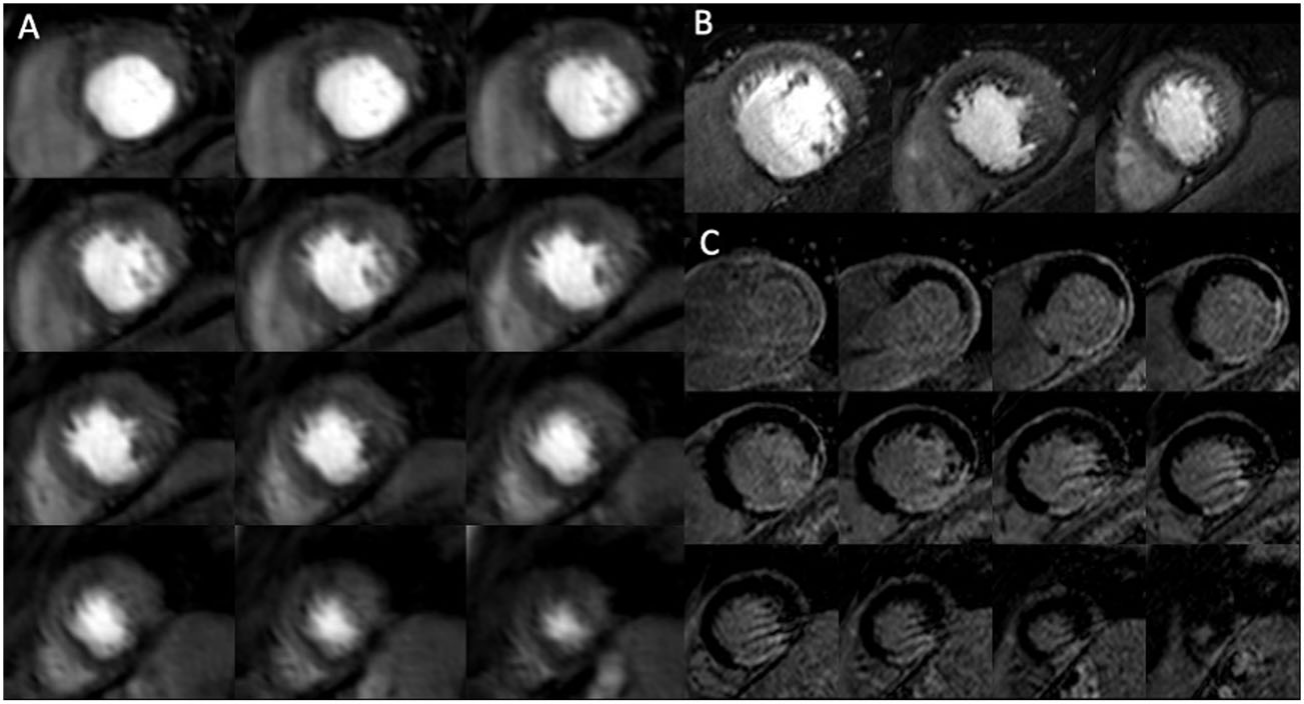
Images at peak myocardial enhancement during adenosine stress perfusion for 3D whole heart (*A*: top to bottom and left to right, from base to apex), 2D high resolution (*B*: left to right from base to apex), and late gadolinium enhancement (LGE) (*C*). Myocardial ischaemic burden was measured as 26.0% (3D whole heart) vs. and 26.2% (2D high resolution). Invasive coronary angiography demonstrated significant LAD disease, mild LCx disease, and occluded RCA. Duke’s jeopardy score was 10, although this does not consider viability as obtained with LGE.

There are several plausible explanations why 2D high resolution identified greater myocardial ischaemic burden compared to 3D myocardial perfusion imaging in our current study (typical cases are illustrated in *Figures 4–6*). Firstly, high resolution imaging accurately identifies the presence of subendocardial ischaemia^4^ and transmural extent of ischaemia through transmural perfusion gradients,^15^ which may not be clearly demarcated with 3D whole heart which has lower spatial resolution. Secondly, a greater number of slices are acquired with 3D whole heart imaging, and hence the total myocardial volume (typically 12 slices, 5 mm thickness) is greater compared to 2D high resolution (typically 3 slices, 8 mm thickness).

Furthermore, while 3D whole heart acquires images of all slices in one defined time point in the systolic phase of cardiac cycle, 2D slices are acquired at different phases of the cardiac cycle, such that the 2D apical slice is acquired typically more closely to late systole and towards early diastole in the cardiac cycle, and hence has a thinner myocardial wall (and hence relatively smaller myocardial volume) compared to a 3D systolic acquisition of the apical slices. Thus, the higher myocardial ischaemia burden [(ischaemia/myocardial volume) × 100] observed with 2D high resolution imaging may relate to the greater detection of subendocardial and transmural extent of ischaemia and a lower total denominator of the total myocardium volume imaged. Another aspect to consider is the point of acquisition in the cardiac cycle for 3D whole heart stress perfusion imaging (diastole vs. systole) which may impact on ischaemia assessment.^16^ Finally, the absence of rest perfusion imaging for both 2D and 3D in this study may have potentially reduced the confidence of reporting ischaemia, as shown previously in a study in which the absence of rest perfusion reduced the confidence of reporting ischaemia, but not the diagnostic accuracy for detection of ischaemia.^17^

Based on this study, we can estimate that using a 2D high resolution stress technique will provide an ∼28% greater myocardial ischaemic burden compared to 3D techniques. The magnitude of difference is important to understand, as different ischaemic burden values can be derived using different CMR methods. As to determine which technique is ultimately correct is challenging, as no formal comparison to a ground truth reference standard for myocardial ischaemia was undertaken in this study. Intuitively, 3D whole heart may considered as more accurate as it has full spatial coverage, but may miss important ischaemia that may be captured from a high resolution technique. Future studies may consider using techniques that allow whole heart spatial coverage and high in plane spatial resolution that may allow detection of subendocardial ischaemia and transmural extent through the entire left ventricle and potentially more accurately delineate the true extent of ischaemia. Such techniques are emerging^18^ although require studies to assess clinical validity, impact on clinical decision making, and long-term outcome.

### Limitations

There are a number of limitations to acknowledge in this study. Firstly, while patients underwent both whole heart and high resolution perfusion imaging in the same scan session, the second contrast injection may have a higher baseline T1 value and potentially impact on the assessment of ischaemia. However, we attempted to minimize this effect by randomizing the order of acquisition of sequences for each patient. Secondly, the assessment of ischaemic burden was undertaken on visual analysis of a series of dynamic perfusion data, which may have introduced variability, although there was very good reproducibility of measurements. Thirdly, a substantial number of cases were excluded from the final analysis, primarily from 3D datasets due to poor image quality from corrupted image readout or poor breath holding. This is an important aspect to consider as despite initial clinical studies that held promise for 3D whole heart with *k-t* acceleration,^19^^,^^20^ such techniques may not be robust enough for clinical practice. Furthermore, the data were not quantified to derive myocardial blood flow, and therefore could be inherently less sensitive for the correct ischaemic burden in patients with multivessel disease.^21^ Finally, the study was performed at a single-centre site using a single-vendor scanner and no external arbiter for assessment for ischaemic burden, such as positron emission tomography, was used.

## Future studies

Methods that use whole heart perfusion imaging combined with high resolution techniques may provide a more accurate assessment of ischaemia. With emerging methods for quantification of absolute myocardial blood flow and with 3D techniques,^22^ future studies could focus on determining the thresholds of ischaemia which are clinically relevant and would allow objective assessments of ischaemic burden. Furthermore, incorporation of novel LGE methods to detect subendocardial scar may allow for more accurate assessment of ischaemic burden, particularly in those patients with multivessel CAD and peri-infarct ischaemia.^23^ Future studies should investigate enhanced coregistration of myocardial perfusion and novel LGE methods and the impact on clinical utility, clinical decision-making, and outcomes in CAD patient.

## Conclusions

2D high resolution and 3D whole heart myocardial perfusion stress CMR are comparable for detection of ischaemia but 2D high resolution measures higher myocardial ischaemic burden compared to a 3D whole heart approach, suggesting that high resolution imaging is more sensitive for the detection of ischaemia. This has important implications for defining significant thresholds for ischaemia.

## Data availability statement

Availability of data and material The datasets used and/or analysed during the current study are available from the corresponding author on reasonable request.
